# Infant morbidity and mortality attributable to prenatal smoking in Chile

**DOI:** 10.26633/RPSP.2017.106

**Published:** 2017-07-20

**Authors:** Jaime Cerda, Claudia Bambs, Claudio Vera

**Affiliations:** 1 Department of Public Health Faculty of Medicine, Pontificia Universidad Católica de Chile Santiago Chile Department of Public Health, Faculty of Medicine, Pontificia Universidad Católica de Chile, Santiago, Chile.; 2 Division of Obstetrics and Gynecology Faculty of Medicine, Pontificia Universidad Católica de Chile Santiago Chile Division of Obstetrics and Gynecology, Faculty of Medicine, Pontificia Universidad Católica de Chile, Santiago, Chile.

**Keywords:** Smoking, maternal exposure, attributable risk, morbidity, infant mortality, Chile, Hábito de fumar, exposición maternal, riesgo atribuible, morbilidad, mortalidad infantil, Chile, Hábito de fumar, exposição maternal, risco atribuível, morbidade, mortalidade infantil, Chile

## Abstract

**Objective:**

*To estimate annual infant morbidity and mortality attributable to prenatal smoking in Chile during 2008−2012*.

**Methods:**

*Population-attributable fractions (PAFs) for several infant outcomes were calculated based on previous study estimates of prenatal smoking prevalence and odds ratios associated with exposure (prenatal smoking relative to non-prenatal smoking). Prenatal smoking–attributable infant morbidity and mortality cases were calculated by multiplying the average annual number of morbidity and mortality cases registered in Chile during 2008–2012 by the corresponding PAF*.

**Results:**

PAFs for 1) births ≤ 27 weeks; 2) births at 28–33 weeks; 3) births at 34–36 weeks; and 4) full-term low-birth-weight infants were 12.3%, 10.6%, 5.5%, and 27.4% respectively. PAFs for deaths caused by preterm-related causes and deaths caused by sudden infant death syndrome were 11.9% and 40.0% respectively. Annually, 2 054 cases of preterm-birth and full-term low-birth-weight (1 in 9 cases), 68 deaths caused by preterm-related causes (1 in 8 cases), and 26 deaths caused by sudden infant death syndrome (1 in 3 cases) were attributable to prenatal smoking.

**Conclusions:**

In Chile, infant morbidity and mortality attributable to prenatal smoking are unacceptably high. Comprehensive individual and population-based interventions for tobacco control should be a public health priority in the country, particularly among female adolescents and young women who will be the mothers of future generations.

## RESUMEN

**Objetivo.:**

Calcular las tasas anuales de morbilidad y mortalidad de lactantes atribuibles al tabaquismo prenatal en Chile entre los años 2008 y 2012.

**Métodos.:**

Se calcularon las fracciones atribuibles a la población (FAP) correspondientes a diferentes resultados de salud en lactantes tomando como base la prevalencia estimativa del tabaquismo prenatal y las razones de posibilidad asociadas a la exposición (tabaquismo prenatal frente a tabaquismo no prenatal) calculadas en estudios previos. Los casos de mortalidad y morbilidad de lactantes atribuibles al tabaquismo prenatal se calcularon multiplicando el promedio anual de casos de morbilidad y mortalidad registrados en Chile entre los años 2008 y 2012 por la FAP correspondiente.

**Resultados.:**

Las FAP para los lactantes nacidos 1) a las 27 semanas o menos de gestación; 2) de 28 a 33 semanas de gestación; 3) de 34 a 36 semanas de gestación; y 4) al término de la gestación, pero con peso bajo al nacer, fueron de 12,3%, 10,6%, 5,5% y 27,4%, respectivamente. Las FAP correspondientes a las muertes por causas relacionadas con el nacimiento prematuro y las muertes por síndrome de muerte súbita del lactante fueron de 11,9% y 40,0%, respectivamente. Al año, 2 054 casos de bebés prematuros y de bebés nacidos con peso bajo al término de la gestación (1 de 9 casos), 68 muertes por causas relacionadas con el período prenatal (1 de 8 casos) y 26 muertes causadas por el síndrome de muerte súbita del lactante (1 de 3 casos) fueron atribuibles al tabaquismo prenatal.

**Conclusiones.:**

En Chile, las tasas de morbilidad y mortalidad de lactantes atribuibles al tabaquismo prenatal son inadmisiblemente altas. En el país, las intervenciones integrales para el control del tabaco a nivel individual y poblacional deben ser una prioridad de salud pública, particularmente en las mujeres adolescentes y jóvenes que serán las madres de las generaciones futuras.

**Objetivo.:**

Estimar a morbidade e a mortalidade infantis anuais atribuíveis ao tabagismo durante a gestação no Chile em 2008–2012.

***Métodos*.:**

As frações atribuíveis populacionais (PAFs) para vários desfechos infantis foram calculadas com base em estimativas de estudos anteriores da prevalência do tabagismo durante a gestação e razões de possibilidades associadas à exposição (tabagismo durante a gestação relativo ao tabagismo fora da gestação). Os casos de morbidade e mortalidade infantis atribuíveis ao tabagismo durante a gestação foram calculados multiplicando-se o número médio anual de casos de morbidade e mortalidade registrados no Chile em 2008–2012 pela PAF correspondente.

**Resultados.:**

As PAFs para 1) recém-nascidos ≤ 27 semanas, 2) recém-nascidos com 28–33 semanas, 3) recém-nascidos com 34–36 semanas e 4) recém-nascidos de termo com baixo peso foram 12,3%, 10,6%, 5,5% e 27,4%, respectivamente. As PAFs para mortes devidas a causas relacionadas à prematuridade e mortes devidas à síndrome de morte súbita do recém-nascido foram 11,9% e 40,0%, respectivamente. Anualmente, 2.054 casos de recém-nascidos prematuros e de termo com baixo peso (1 em 9 casos), 68 mortes devidas a causas relacionadas à assistência pré-natal (1 em 8 casos) e 26 mortes devidas à síndrome de morte súbita do recém-nascido (1 em 3 casos) foram atribuíveis ao tabagismo durante a gestação.

**Conclusões.:**

No Chile, a morbidade e a mortalidade infantis atribuíveis ao tabagismo durante a gestação são inadmissivelmente elevadas. Amplas intervenções individuais e populacionais para o controle do tabagismo devem ser uma prioridade de saúde pública no país, sobretudo em adolescentes e jovens que serão mães das futuras gerações.

Prenatal smoking is a known risk factor for infant morbidity and mortality, including its role in the genesis of low birth weight, respiratory complications, and sudden infant death (1). The prevalence of cigarette smoking among Chilean women is the highest in the Americas region (2), especially among those of reproductive age (42.6% among women 15–24 years old and 44.3% among women 25–44 years old) (3). The average cigarette consumption among women 25–44 years old is 10.0 cigarettes per day (3). Looking toward the future, the situation is worrisome: according to the Global School Health Survey (2013), the percentage of female students 13–15 years old in Chile who used any tobacco products on one or more days during the past 30 days is 27.8%, the highest in the Americas (4). A study conducted in 2007 by Mallol et al. in a low SES^3^ area in Santiago, the capital city, showed that 1) estimated overall prenatal smoking prevalence was 28.0%; 2) 65.7% of pregnant women were exposed to secondhand tobacco smoke inside their homes; and 3) newborns of mothers who smoked during pregnancy had a lower birth weight than newborns whose mothers did not smoke during pregnancy (3 424 g versus 3 336 g) (5). Two studies conducted in Chile, by Telgie (2007) and Pérez-Franco & Raffo (2015), found an estimated prenatal smoking prevalence of 31.5% (6) and 36.4% (7) respectively. These prevalence estimates support the idea that prenatal smoking is a major public health problem in Chile, affecting 1 out of every 3–4 pregnant women. Estimates for morbidity and mortality attributable to smoking have been used to 1) quantify the effect of smoking at the population level, 2) monitor it over time, and 3) determine priorities for public health actions in different countries (8). In Chile, the Department of Health Economics of the Ministry of Health and the Institute for Clinical Effectiveness and Health Policy estimated the annual number of deaths attributable to cigarette smoking among the general population in 2014 at 16 532 deaths, equivalent to 18.5% of overall mortality. Annual direct costs attributable to smoking are equivalent to 0.8% of the Chilean gross domestic product and 11.5% of the annual health budget (9). However, these estimates have focused mainly on selected diseases affecting adults. Estimates for infant outcomes attributable to prenatal smoking are scarce worldwide (10) and are not available in Chile. This information could be useful, as smoking during pregnancy is preventable and national policy initiatives can be supported by such data. Thus, the objective of this study was to estimate the annual infant morbidity and mortality attributable to prenatal smoking in Chile during 2008−2012.

## MATERIALS AND METHODS

This study was based on estimates of population-attributable fractions (PAFs), using secondary data provided by prevalence and cohort studies.

Information on overall prenatal smoking prevalence in Chile was obtained from the study carried out by Mallol et al. (2007) in which a random sample of 400 Chilean mothers from two districts in Santiago were interviewed by a pediatrician during their hospitalization in their first few days postpartum (5). The estimated prenatal smoking prevalence found in that study was 28.0% (95% confidence interval: 23.8%–32.6%).

Data for overall and cause-specific infant morbidity and mortality in Chile during 2008–2012 were obtained from the Department of Statistics and Health Information of the Ministry of Health (11). Infant morbidity was calculated for singleton preterm births (< 37 weeks) and full-term low-birth-weight (≥ 37 weeks and < 2 500 g). The analysis of infant mortality (i.e., deaths in children under one year) included the following conditions, based on ICD-10^4^ criteria and coding: preterm-related causes (K550, P000, P010, P011, P015, P020, P021, P027, P070–P073, P102, P220–P229, P250–P279, P280, P281, P360–P369, P520–P523, P77) and sudden infant death syndrome (R95) (12) (Table 1).

**TABLE 1.** ICD-10^a^ codes for diseases and related health problems included in estimates of infant mortality, Chile, 2008–2012

ICD-10 code	Diseases and related health problems
K550	Acute vascular disorders of intestine
P000	Fetus and newborn affected by maternal hypertensive disorders
P010	Fetus and newborn affected by incompetent cervix
P011	Fetus and newborn affected by premature rupture of membranes
P015	Fetus and newborn affected by multiple pregnancy
P020	Fetus and newborn affected by placenta previa
P021	Fetus and newborn affected by other forms of placental separation and hemorrhage
P027	Fetus and newborn affected by chorioamnionitis
P070−P073	Disorders related to short gestation and low birth weight, not elsewhere classified
P102	Intraventricular hemorrhage due to birth injury
P220−P229	Respiratory distress of newborn
P250−P279	Interstitial emphysema and related conditions originating in the perinatal period
	Pulmonary hemorrhage originating in the perinatal period
	Chronic respiratory disease originating in the perinatal period
P280	Primary atelectasis of newborn
P281	Other and unspecified atelectasis of newborn
P360−P369	Bacterial sepsis of newborn
P520−P523	Intraventricular (nontraumatic) hemorrhage, grade 1, of fetus and newborn
	Intraventricular (nontraumatic) hemorrhage, grade 2, of fetus and newborn
	Intraventricular (nontraumatic) hemorrhage, grade 3, of fetus and newborn
	Unspecified intraventricular (nontraumatic) hemorrhage of fetus and newborn
P77	Necrotizing enterocolitis of fetus and newborn
R95	Sudden infant death syndrome

a International Classification of Diseases, 10^th^ revision.

***Source:*** Prepared by the authors based on the ICD-10.

Infant morbidity and mortality attributable to prenatal smoking were calculated by multiplying the average annual number of cases registered during 2008–2012 by the corresponding PAF, using Formula #1:

*PAF = pd[(ARR–1)/ARR]* (13)

where pd = the proportion of cases exposed to prenatal smoking and ARR = adjusted relative risk for each outcome associated with the exposure (prenatal smoking relative to non-prenatal smoking). Adjusted odds ratios (AORs) were used as estimates of ARRs. The AORs for infant morbidity and mortality were obtained from the study done by Dietz et al. (12) and were as follows: AOR = 1.5 for gestation ≤ 27 weeks; AOR = 1.4 for gestation 28–33 weeks; AOR = 1.2 for gestation 34–36 weeks; AOR = 2.3 for gestation ≥ 37 weeks and birth weight < 2 500 g; AOR = 1.5 for preterm-related causes; and AOR = 2.7 for sudden infant death syndrome. For each of the selected outcomes, the proportion of cases exposed to prenatal smoking (pd) was estimated using the four-step procedure described below (with more details provided in Supplementary Material Annex 1).

### Step 1.

Using the data provided by Dietz et al.’s study (12), the number of cases and controls exposed to prenatal smoking and the number of cases and controls non-exposed to prenatal smoking were calculated.

### Step 2.

Using the data derived from Step 1, the crude relative risk (CRR) (prenatal smoking relative to non-prenatal smoking) was calculated using the following formula: CRR = (cases exposed/overall exposed)/(cases non-exposed/overall non-exposed).

### Step 3.

Based on the overall number of cases and controls registered in Chile, the overall prenatal smoking prevalence in Chile (i.e., 28.0%) (5), and the CRR calculated in Step 2, the theoretical number of cases and controls exposed to prenatal smoking in Chile was calculated. This was accomplished using two equations: Equation #1 (theoretical cases exposed + theoretical cases non-exposed) = overall cases registered in Chile, and Equation #2 (theoretical cases exposed/theoretical overall exposed)/(theoretical cases non-exposed/theoretical overall non-exposed) = CRR (calculated in Step 2).

### Step 4.

The theoretical proportion of cases exposed to prenatal smoking in Chile (pd) was calculated using the following formula: pd = (theoretical cases exposed/overall cases registered in Chile).

Finally, given that Levin’s formula for calculating PAFs ([p(RR–1)]/[p(RR–1)+1]) (13) is widely used in epidemiological textbooks and studies, all of the estimates were also calculated using this formula and compared to results obtained in the initial approach based on Formula #1. In Levin’s formula, p = prenatal smoking prevalence and RR = relative risk for each outcome associated with the exposure (prenatal smoking relative to non-prenatal smoking).

PAFs for each selected outcome were presented as percentages. All statistical analyses were conducted using Microsoft Excel for Windows (Microsoft, Redmond, Washington, United States). The Ethics and Security in Research Coordination Office at Pontificia Universidad Católica de Chile reviewed the protocol and declared it exempt from Institutional Review Board evaluation.

## RESULTS

### Infant morbidity attributable to prenatal smoking

In Chile, 1 214 831 singletons were born during 2008–2012 (97.9% of overall live births), and 94 581 (7.8%) were either preterm or full-term low-birth-weight (18 917 cases per year). The PAFs for prenatal smoking were 12.3% for gestational age ≤ 27 weeks, 10.6% for gestational age 28–33 weeks, 5.5% for gestational age 34–36 weeks, and 27.4% for gestational age ≥ 37 weeks and birth weight < 2 500 g (Table 2). The overall morbidity attributable to prenatal smoking was 2 054 cases per year; nearly half of which corresponded to the outcome gestational age ≥ 37 weeks and birth weight < 2 500 g (977 cases per year), followed by the outcome gestational age 34–36 weeks (610 cases per year) (Table 3).

**TABLE 2.** Prenatal smoking population-attributable fractions (PAFs) for infant morbidity and mortality, Chile, 2008–2012

Outcome	Theoretical proportion of cases exposed (pd)(%)	Adjusted odds ratio (AOR)	PAF^a^ (%)
Infant morbidity Gestational age			
≤ 27 weeks	36.8	1.5	12.3
28–33 weeks	37.2	1.4	10.6
34–36 weeks	33.0	1.2	5.5
≥ 37 weeks and < 2 500 g	48.4	2.3	27.4
Infant mortality			
Preterm-related causes^b^	35.8	1.5	11.9
Sudden infant death syndrome^c^	63.5	2.7	40.0

a pd[(AOR–1)/AOR].

b International Classification of Diseases, 10th revision (ICD-10) codes K550, P000, P010, P011, P015, P020, P021, P027, P070–P073, P102, P220–P229, P250–P279, P280, P281, P360–P369, P520–P523, and P77.

c ICD-10 code R95.

***Source:*** Prepared by the authors based on the study results.

**TABLE 3.** Infant outcomes (morbidity and mortality) attributable to prenatal smoking, Chile, 2008–2012

Outcome	Overall cases per year	Cases attributable to prenatal smoking per year
Infant morbidity Gestational age		
≤ 27 weeks	937	115
28–33 weeks	3 325	352
34–36 weeks	11 091	610
≥ 37 weeks and < 2 500 g	3 564	977
Total	18 917	2 054
Infant mortality		
Preterm-related causes^a^	571	68
Sudden infant death syndrome^b^	64	26
Total	635	94

a International Classification of Diseases, 10^th^ revision (ICD-10) codes K550, P000, P010, P011, P015, P020, P021, P027, P070–P073, P102, P220–P229, P250–P279, P280, P281, P360–P369, P520–P523, and P77.

b ICD-10 code R95.

***Source:*** Prepared by the authors based on the study results.

### Infant mortality attributable to prenatal smoking

In Chile, 9 527 infants died during 2008–2012, for an overall annual infant mortality rate of 7.7 per 1 000 newborns. Of those 9 527 infants, 2 856 died from preterm-related causes and 319 died from sudden infant death syndrome. Those two causes combined represented one-third of all infant deaths registered in Chile during the study period (3 175 out of 9 527). The PAFs for prenatal smoking were 11.9% for preterm-related causes and 40.0% for sudden infant death syndrome (Table 2). The overall mortality for these outcomes attributable to prenatal smoking was 94 deaths per year: 68 deaths per year for preterm-related causes and 26 deaths per year for sudden infant death syndrome (Table 3).

### Alternative approach for PAF estimation using Levin’s formula

Using Levin’s formula, PAFs for prenatal smoking were 12.3% for gestational age ≤ 27 weeks, 10.1% for gestational age 28–33 weeks, 5.3% for gestational age 34–36 weeks, and 26.7% for gestational age ≥ 37 weeks and birth weight < 2 500 g. The overall mortality attributable to prenatal smoking was 1 989 cases per year (65 fewer cases than was found using Formula #1). On the other hand, PAFs for prenatal smoking were 12.3% for preterm-related causes and 32.2% for sudden infant death syndrome. The overall prenatal smoking attributable mortality for these outcomes was 91 deaths per year (3 cases less than was found using Formula #1).

## DISCUSSION

The estimates found in this study for Chile showed that 1 in 9 cases of singleton preterm or full-term low-birth-weight newborns, 1 in 8 infant deaths caused by preterm-related causes, and 1 in 3 deaths caused by sudden infant death syndrome are attributable to prenatal smoking. To the best of the authors’ knowledge, this is the first report on infant morbidity and mortality in Chile attributable to prenatal smoking.

The validity of these results relies on the assumption that prenatal smoking is a sufficient cause for the selected outcomes, as well as on the methods used to estimate the PAFs. In addition, the use of the best available estimates of overall prenatal smoking prevalence and relative risks of infant morbidity and mortality for prenatal smoking relative to prenatal non-smoking was critical for calculating reliable PAF calculations. In Chile, four different studies have estimated prenatal smoking prevalence. The study conducted by Mallol et al. (5) evaluated tobacco consumption in a sample of 400 pregnant women from a low-SES area in Santiago during 2007. Women were interviewed by a pediatrician during hospitalization in their first few days postpartum, using a comprehensive questionnaire about smoking, which included questions about occasional cigarette consumption. A total of 28% of the mothers reported having smoked during their recent pregnancy, and 68.0% reported that they smoked before pregnancy (45.1% daily and 22.9% occasionally). This study could have overestimated prenatal smoking prevalence, given that smoking prevalence among nonpregnant women aged 25–44 in Chile was 44.3%, according to the Chilean National Health Survey 2009–2010 (3). Nevertheless, two other studies reported even higher prenatal smoking prevalence: Telgie 2007 (31.5%) (6) and Pérez-Franco & Raffo 2015 (36.4%) (7). On the other hand, the Tenth National Study of Drugs in the General Population of Chile (*Décimo Estudio Nacional de Drogas en Población General de Chile*) estimated a prenatal smoking prevalence of 4.8%, using a single question (“*In your most recent pregnancy, did you smoke tobacco regularly, say, almost every day?”*) in a sample of 6 937 pregnant women (14). The question was self-administered and women were asked about their smoking habit in their last pregnancy, which occurred at least three years before for 80% of the respondents, creating the potential for recall bias and underestimation of the true prevalence of prenatal smoking in Chile, given the epidemic of smoking among Chilean women of reproductive age. The authors of this study decided to include the prenatal smoking prevalence reported by Mallol et al. (5) in the calculation of PAFs because it seems methodologically less biased and its magnitude appears to be realistic for a country with the highest smoking prevalence in the Americas region. In addition, Chile exhibits a social gradient in smoking prevalence, with significantly lower prevalence of smoking among low-SES groups (3). Therefore, the prenatal smoking prevalence reported by Mallol et al., which was based on a sample of women from a low-SES area, may be a conservative estimate of the true national prevalence of prenatal smoking in Chile.

### Relative risk of infant morbidity and mortality for prenatal smokers

For this analysis, the authors decided to use the AORs reported by Dietz et al. in 2010 (12), which provide important benefits, including 1) an expanded definition of death from preterm delivery, going beyond the use of ICD-10 codes for disorders related to short gestation and low birth weight; 2) number of infant deaths limited to those for which prenatal smoking has an established causal effect; and 3) ORs adjusted by a set of known confounders, such as maternal age, education, race/ethnicity, marital status, parity, and infant gender, among others (15). Consequently, the AORs used in this study are the current best available secondary evidence for performing an unbiased estimation of infant morbidity and mortality attributable to prenatal smoking.

This analysis could have used Levin’s formula for calculating PAFs ([p(RR–1)]/[p(RR–1)+1]), given that it is widely used in epidemiological textbooks and studies. However, the use of ARRs in that formula has been recognized as a common error, as the formula is not considered valid where there is confounding of the exposure-disease association (13). Therefore, the authors of this study decided to use an alternative formula to calculate the PAFs: pd[(ARR–1)/ARR)] (Formula #1). Formula #1 produces an internally valid estimate when confounding exists and when, as a result, ARRs must be used. This alternative expression requires either knowing or estimating pd (the proportion of cases exposed to prenatal exposure) for Chilean diseases and related health problems. In this study, a four-step procedure was used to estimate several pds. This procedure was based on the assumption that CRRs derived from Dietz at al.’s study are valid proxies for Chilean CRRs, just as it was assumed that ARRs provided by Dietz et al.’s study are valid proxies for Chilean adjusted risks (justifying their use in the PAF formula). As a complementary analysis, PAFs were also estimated using Levin’s formula (including AORs from Dietz’s study), which generated similar results: 2 054 versus 1 989 cases of infant morbidity and 94 versus 91 cases of infant mortality attributable to prenatal smoking, which supports the validity of the results.

Evidence shows that smoking prevalence at the population level can be reduced through comprehensive and sustained tobacco control strategies. The WHO Framework Convention on Tobacco Control (WHO FCTC), through its MPOWER^5^ program (16), provides a comprehensive guide on tobacco control. Several advances in public health have been made since Chile signed and ratified the WHO FCTC in 2005, including 1) the establishment of smoke-free environments in closed public spaces, 2) an increase in regulations on tobacco marketing and advertising, and 3) an increase in tobacco taxes. However, little progress has been made on health care insurance for smoking cessation services, including those for pregnant women who smoke. Currently, most pregnant women in Chile are served by the public health system, which offers assessment of smoking status and brief counseling for smoking cessation as part of prenatal care (17). However, time constraints and a lack of training in tobacco cessation counseling limits the provision of these services by health care providers (18). Smoking cessation interventions beyond brief counseling are currently not covered by the Chilean health system, public or private. This is particularly worrisome given the extremely high prevalence of cigarette smoking among Chilean women and adolescents. The reasons behind this epidemic are beyond the scope of this article but might include the challenges Chile faces as a country in “stage 3” of its smoking epidemic. According to the model for cigarette epidemics developed by Lopez et al., stage 3 is the period in which smoking prevalence is stable or decreasing but smoking-attributed mortality is increasing rapidly in both women and men (19).

There are also potential psychological and social explanations for the fact that Chilean women are at the top in terms of global tobacco consumption rates. Data from the Chilean National Health Survey 2009–2010 show that 21.7% of women 15–24 years old and 27.9% of women 25–44 years old have symptoms of depression, and a high prevalence of symptoms for other psychological domains, such as hostility, stress and anxiety (3). Women have been the target of aggressive marketing strategies promoting Western social values, and socially disadvantaged groups may be particularly vulnerable to the campaigns (20).

Currently, there are several interventions with proven efficacy in supporting pregnant women to stop smoking (21). As this study suggests, if Chilean prenatal smoking prevalence decreases, a significant number of cases of stillbirths, infant morbidity, and infant mortality could be potentially avoided. Given that the estimates provided here did not include the contribution of passive smoking to stillbirths and infant outcomes, these results should be considered conservative estimates of the burden of infant morbidity and mortality attributable to prenatal smoking.

Since May 2016, the warning on tobacco packaging in Chile has included, for the first time, messages and images focused on pregnant women and infants. One image shows the belly of a pregnant woman surrounded by cigarette smoke and the following message: “*Warning: If you smoke, you intoxicate your son/daughter.”* Another image shows a premature infant with a feeding tube and the following message: “*Warning: If you smoke, you make him/her sick.”* The authors believe this will help raise awareness about the risk of prenatal smoking while other public health and clinical efforts are being coordinated to 1) reduce the impact of smoking on future generations of Chileans and 2) increase the benefits to women’s health once smokers quit.

### Strengths and limitations

This study has the strengths and limitations of most studies that estimate smoking-attributable morbidity and mortality using PAFs. Strengths include estimates based on the best secondary data available for Chilean prenatal smoking prevalence and infant risks associated with prenatal smoking, providing validity to the results. On the other hand, there were several methodological limitations, including: 1) the set of diseases and health conditions included as “preterm-related disorders” may not be exhaustive / fully representative; 2) the possibility that prenatal smoking prevalence was underestimated, given the social stigma associated with it; and 3) the fact that most infant diseases and health-related conditions are multifactorial.

### Conclusions

This study estimated annual morbidity and mortality attributable to prenatal smoking during 2018−2012 in Chile, the country with the highest smoking prevalence in the Americas region. This information could be used in the public policy formulation process in order to minimize the deleterious effects of prenatal smoking on infants.

### Funding.

Claudia Bambs receives funding from the Advanced Center for Chronic Diseases (ACCDiS), Fondap project 15130011.

### Disclaimer.

Authors hold sole responsibility for the views expressed in the manuscript, which may not necessarily reflect the opinion or policy of the *RPSP/PAJPH* or the Pan American Health Organization (PAHO).

## Footnotes

3 Socioeconomic status.

4 World Health Organization (WHO) International Classification of Diseases, 10th revision.

5 Six measures to facilitate country-level implementation of the WHO FCTC: 1) Monitor tobacco use and prevention policies; 2) Protect people from tobacco smoke; 3) Offer help to quit tobacco use; 4) Warn people about tobacco; 5) Enforce bans on advertising, promotion and sponsorship; and 6) Raise the price of tobacco.
